# Deep learning approach towards accurate state of charge estimation for lithium-ion batteries using self-supervised transformer model

**DOI:** 10.1038/s41598-021-98915-8

**Published:** 2021-10-01

**Authors:** M. A. Hannan, D. N. T. How, M. S. Hossain Lipu, M. Mansor, Pin Jern Ker, Z. Y. Dong, K. S. M. Sahari, S. K. Tiong, K. M. Muttaqi, T. M. Indra Mahlia, F. Blaabjerg

**Affiliations:** 1 Department of Electrical Power Engineering, COE, Universiti Tenaga Nasional, 43000 Kajang, Malaysia; 2 Department of Electrical, Electronic and Systems Engineering, Universiti Kebangsaan Malaysia, 43600 Bangi, Malaysia; 3 Institute of Sustainable Energy, Universiti Tenaga Nasional, 43000 Kajang, Malaysia; 4 School of Electrical Engineering and Telecommunications, UNSW, Kensington, NSW 2033 Australia; 5 Department of Mechanical Engineering, COE, Universiti Tenaga Nasional, 43000 Kajang, Malaysia; 6 School of Electrical, Computer and Telecommunications Engineering, University of Wollongong, Wollongong, NSW 2522 Australia; 7 Present Address: Centre of Green Technology, Faculty of Engineering and Information Technology, University of Technology Sydney, Ultimo, NSW 2007 Australia; 8 Department of Energy Technology, Aalborg University, 9220 Aalborg, Denmark

**Keywords:** Energy science and technology, Engineering, Mathematics and computing

## Abstract

Accurate state of charge (SOC) estimation of lithium-ion (Li-ion) batteries is crucial in prolonging cell lifespan and ensuring its safe operation for electric vehicle applications. In this article, we propose the deep learning-based transformer model trained with self-supervised learning (SSL) for end-to-end SOC estimation without the requirements of feature engineering or adaptive filtering. We demonstrate that with the SSL framework, the proposed deep learning transformer model achieves the lowest root-mean-square-error (RMSE) of 0.90% and a mean-absolute-error (MAE) of 0.44% at constant ambient temperature, and RMSE of 1.19% and a MAE of 0.7% at varying ambient temperature. With SSL, the proposed model can be trained with as few as 5 epochs using only 20% of the total training data and still achieves less than 1.9% RMSE on the test data. Finally, we also demonstrate that the learning weights during the SSL training can be transferred to a new Li-ion cell with different chemistry and still achieve on-par performance compared to the models trained from scratch on the new cell.

## Introduction

The transportation and electricity production sectors account for more than 50% of total green-house gas emissions^1^ as both rely on fossil fuels as the energy source. Promising solutions include the electrification of the transportation industry and the decarbonization of electrical grids^2,3^. However, the mass adoption of electric vehicles and renewable energy remains low due to the high adoption cost which can be attributed to the Li-ion batteries^4^. A major challenge in Li-ion batteries research is the state of charge (SOC) estimation which signifies the amount of charge left in a Li-ion battery cell^5^. Accurate SOC estimation allows the Li-ion battery cells to be used to its maximum potential before disposal, resulting in tremendous cost savings in the manufacturing and adoption costs^6^. Nevertheless, it is a notoriously hard to quantify SOC as it cannot be practically measured by sensors outside laboratory environment with existing sensor technologies^7^.

Two most common approaches used in SOC estimation are the model-based and data-driven approaches^8^. Model-based approach leverages on an in-depth understanding of domain knowledge such as the internal chemical reaction in the cell, electrical properties of the components used to model them and complex mathematical equations to model the SOC^9^. Prominent model-based techniques include the Sliding Mode Observer^10^, Luenberger Observer^11^, Kalman filters^12^ Electrochemical Model^13^, Equivalent Circuit Model^14^, Electrochemical Impedance Model^15^. While model-based approach can result in reliable and accurate models, it requires an extensive domain knowledge, rigorous feature engineering, and relatively long development time^16^. Apart from that, model-based approach also does not scale well across battery cells different chemistry. As a result, alterations in cell chemistry requires a re-development of the model^17^. Additionally, model-based approach also does not account for anomalies in cells such as manufacturing inconsistencies, unpredictable operating conditions, cell degradation, and so forth^18^. Due to these shortcomings, more researchers are shifting their attention to using the data-driven approach for SOC estimation. In this approach, the SOC is directly modeled from observable signals such as voltage, current and temperature of the Li-ion battery cell sampled over diverse operating conditions across different cell chemistry and manufacturers^19^. There are various methods in data-driven SOC estimation such as artificial neural network^20^, support vector machine^21^, extreme learning machine^22^ Gaussian process regression^23^, wavelet neural network^24^, nonlinear autoregressive with exogenous input neural network^25^, optimized neural network^26^ and fuzzy logic^27^ to name a few. One method that has been gaining traction lately is the use of a data-driven technique known as deep learning (DL)^28^. DL has great potential for SOC estimation due to its powerful capability to learn any function given the right data according to the universal approximation theorem^29^. In essence, DL can be used to directly approximate the relationship between the measurable cell signals (voltage, current, temperature) and the SOC with no additional processing such as using adaptive filters^30^. This eliminates the needs of manual feature engineering which can take a considerable amount of time and expert domain knowledge and still produce accurate SOC estimation results^31^. Pioneering works by authors^32,33^ introduce long short-term memory (LSTM) and deep neural network DNN to directly estimate SOC from cell voltage, current and temperature with no additional filters. The proposed model achieved lowest MAE of 2.17% over a varying ambient temperature dataset. Authors in^34,35^ proposed the utilization of gated recurrent unit (GRU) model to directly estimate SOC over a wide ambient temperature range with a RMSE of 3.5% under untrained temperature. There are also authors who proposed deep convolutional models such as in^36,37^ with approximately less than 3% MAE on untrained data. Nonetheless, there are works on hybridizing convolutional and recurrent models such as^35,38^ with under 2% RMSE on varying ambient temperature.

However, there are several research gaps with the existing DL methods for SOC estimation. These research gaps are the primary motivations of the proposal in this article. Firstly, all the cited works uses the supervised learning (SL) scheme to train the models which is known to require massive amount of data to accomplish^39^. Even in the scenarios where adequate data is available the training time for DL models typically takes many hours or days to complete^32^. Secondly, the models that are trained on one cell chemistry do not apply to other cell chemistry. Even though preliminary work indicates that transfer learning is possible^40^, further tests are still required to verify its accuracy if it applies to more cells with differing chemistry. In most cases a model that is trained on one Li-ion battery cell data does not generalize well across another cell and may require re-training of the model from scratch. Thirdly, most DL models use the recurrent DL architecture which may prove to work well with sequence data such as the SOC but are hard and slow to train^41^. Recurrent models also do not leverage on the parallel GPU computation that could significantly improve training time^25^. Lastly, even though recurrent architectures such as the LSTM or GRU can handle long sequences well, they are still susceptible to vanishing gradient for longer sequences^42^. Due to these limitations, recurrent models are generally superseded by another architecture known as the Transformer in many domains such as computer vision^43^ and natural language processing^44,45^. In short, the primary motivations for the proposal in this article are (i) shortcomings of the SL training framework, (ii) Inadequate validation and testing of transfer learning capabilities in DL models, (iii) shortcomings of the DL recurrent architectures and (iv) Emergence and success of the Transformer DL model in other domains.

In this article, we introduce a new DL architecture for SOC estimation known as the Transformer. Apart from that, we propose a training framework that leverages on self-supervised learning (SSL)^39,46^ and make it possible to train the Transformer on scarce amounts of Li-ion data in a short time and achieve higher estimation accuracy compared to models trained with conventional fully-supervised method. With the proposed framework, we demonstrate that the learned parameters from one cell can be transferred to another by fine-tuning the model on little data with very short training time (approximately 30 min on a GPU). This proposed framework also incorporates various recent DL techniques such as using Ranger optimizer with learning rate finder, time series data augmentation, and Log-Cosh loss function to boost the accuracy of the Transformer. Finally, we conclude the study by comparing and validating the performance of our proposed model to other recent DL architectures for SOC estimation. The key contributions of this study are as follows:

- We introduce the transformer DL architecture for end-to-end SOC estimation with no feature engineering or adaptive filtering.
- We propose the SSL training framework to train the proposed architecture in a very short amount of time and achieves improved estimation accuracy compared to conventional training framework.
- The proposed model’s parameters are transferable to a different cell type and requires only five training epochs to achieve RMSE ≤ 2.5%.
- The SSL training framework enables the proposed model to be re-trained with as few as 20% of the total training data and still achieve lower error compared to the models that do not use the SSL framework.
- We evaluate and validate the performance of the proposed model to other state-of-the-art DL architectures for SOC estimation.

## Results

In the first two subsections, we highlight the estimation accuracy of the proposed model trained at room temperature and varying ambient temperatures and compare the estimation robustness against other DL models. In the subsequent sections, we study the influence of pre-training on the model and show that the pre-trained model outperforms the non-pre-trained models in estimation accuracy and convergence time. Next, we highlight that the learned weights of the pre-trained model can be transferred to perform estimation on a different cell with different chemistry with short re-training or fine-tuning. In all experiments, the proposed Transformer model was benchmarked against other widely used DL models for SOC estimation of various architecture types. The hyperparameter combinations of all comparison models were chosen to be as close as possible to the original publication. If that is infeasible, we adopted the hyperparameter configurations that is widely accepted or used and has proven to work well on many occasions. All common hyperparameters such as sliding window value, input output units, learning rate, batch size, training epochs were held constant. All comparison models were trained with the SL training framework and only the proposed Transformer model was trained with the SSL training framework under the same battery materials and EV drive cycle dataset.

### Estimation accuracy under constant ambient temperature

**Figure 1:**
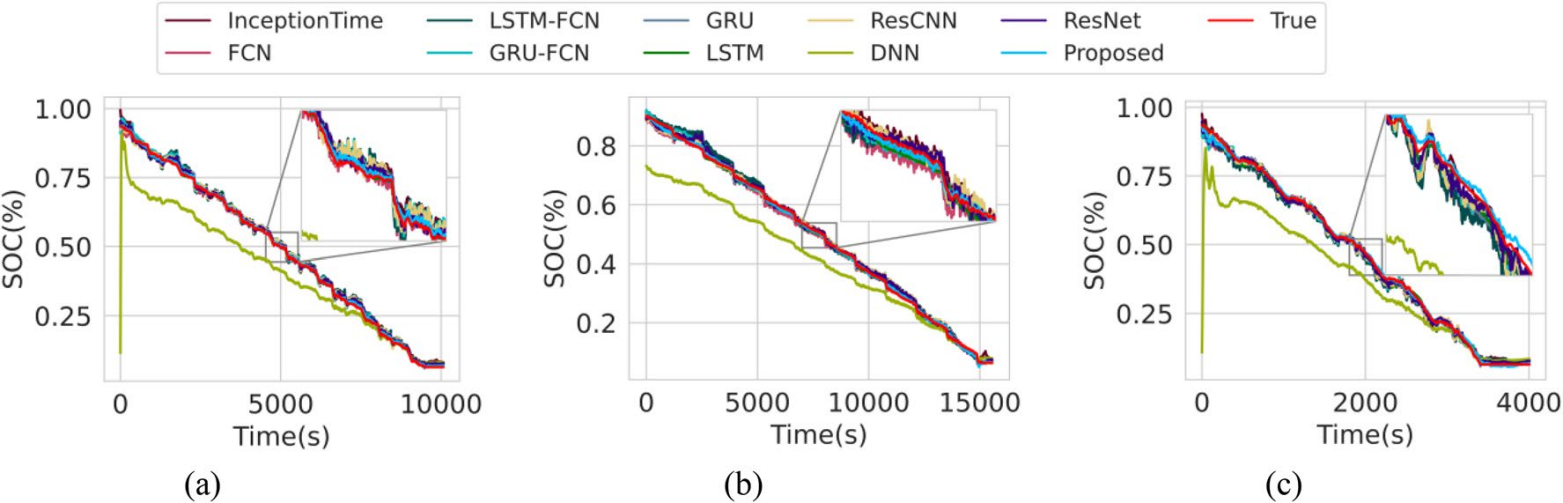
SOC estimation at room temperature. (**a**) LA92 drive cycle at 25 °C. (**b**) UDDS drive cycle at 25 °C. (**c**) US06 drive cycle at 25 °C.

In this section, we demonstrate the estimation accuracy and efficacy of the proposed model based on data sampled at room temperature. The error metrics of all models are tabulated in Table 1, sorted in ascending order of the test error. We found that the proposed model performance on SOC estimation accuracy of the RMSE in terms of training, validation and testing are 1.1087%, 0.8661%, 0.9056% and the MAE are 0.3289%, 0.4059%, and 0.4459%, respectively. It is observed that the proposed model outperforms all other models on the test dataset of RMSE and MAE tabulated in Table 1. For comparison, a baseline Transformer model that was trained using conventional training framework is included. Results indicate that the baseline model only scores abysmally compared to other models except the DNN, suggesting that the training framework plays a pivotal role in the robust performance of the proposed model. We observe that the recurrent models (GRU and LSTM) outperformed their convolutional and hybrid counterpart, which is not surprising as the recurrent models are specifically designed to handle sequence data well. Both the GRU and LSTM were configured to use single hidden layer with 100 neurons. Despite the compromise in estimation accuracy, convolutional models may be advantageous in training complexity compared to the recurrent models, as they are relatively easier to train and could better utilize GPU parallelization. The Resnet model is based on the Residual Network architecture^47^ adapted to work sequential data^48^. Inception Time is based on the implementation in^49^ which has shown superior benchmark performance in sequential data. ResCNN consist of convolutional neural network with Residual blocks as inputs. FCN consist of only convolution operation with no pooling operations has shown promising performance in^50^. GRU-FCN and LSTM-FCN are the hybrid models combining recurrent and convolutional models to obtain the advantages of both. The estimation plot on the test dataset consisting of US06, LS92 and UDDS drive cycle is shown in Fig. 1.

**Table 1:** SOC estimation accuracy comparison on various DL models on the train, validation, and test dataset at constant ambient temperature.

Name	Arch. type	Train error (%)		Valid. error (%)		Test error (%)	
		RMSE	MAE	RMSE	MAE	RMSE	MAE
**Proposed**	**Transformer**	1.1087	0.3289	0.8661	0.4059	0.9056	0.4459
GRU	Recurrent	1.5028	0.4461	0.9852	0.3624	1.0686	0.4877
LSTM	Recurrent	1.6951	0.5404	1.0699	0.4370	1.1381	0.5341
Resnet	Convolutional	1.5523	0.5647	1.1395	0.5924	1.3349	0.7859
ResCNN	Convolutional	1.6121	0.6134	1.1879	0.6548	1.3454	0.8031
InceptionTime	Convolutional	1.8177	0.7538	1.3079	0.7259	1.4404	0.8202
GRU-FCN	Hybrid	1.7138	0.5978	1.3405	0.6939	1.4477	0.8228
FCN	Convolutional	1.8635	0.7685	1.2912	0.6732	1.5555	0.9642
LSTM-FCN	Hybrid	1.7619	0.7611	1.4123	0.8218	1.7771	1.1954
Baseline	Transformer	3.8265	2.2195	2.4050	1.2735	3.6116	2.7453
DNN	Feedforward	11.0576	8.6442	10.4213	8.5476	10.1347	8.2181

### Estimation accuracy under varying ambient temperatures

**Figure 2:**
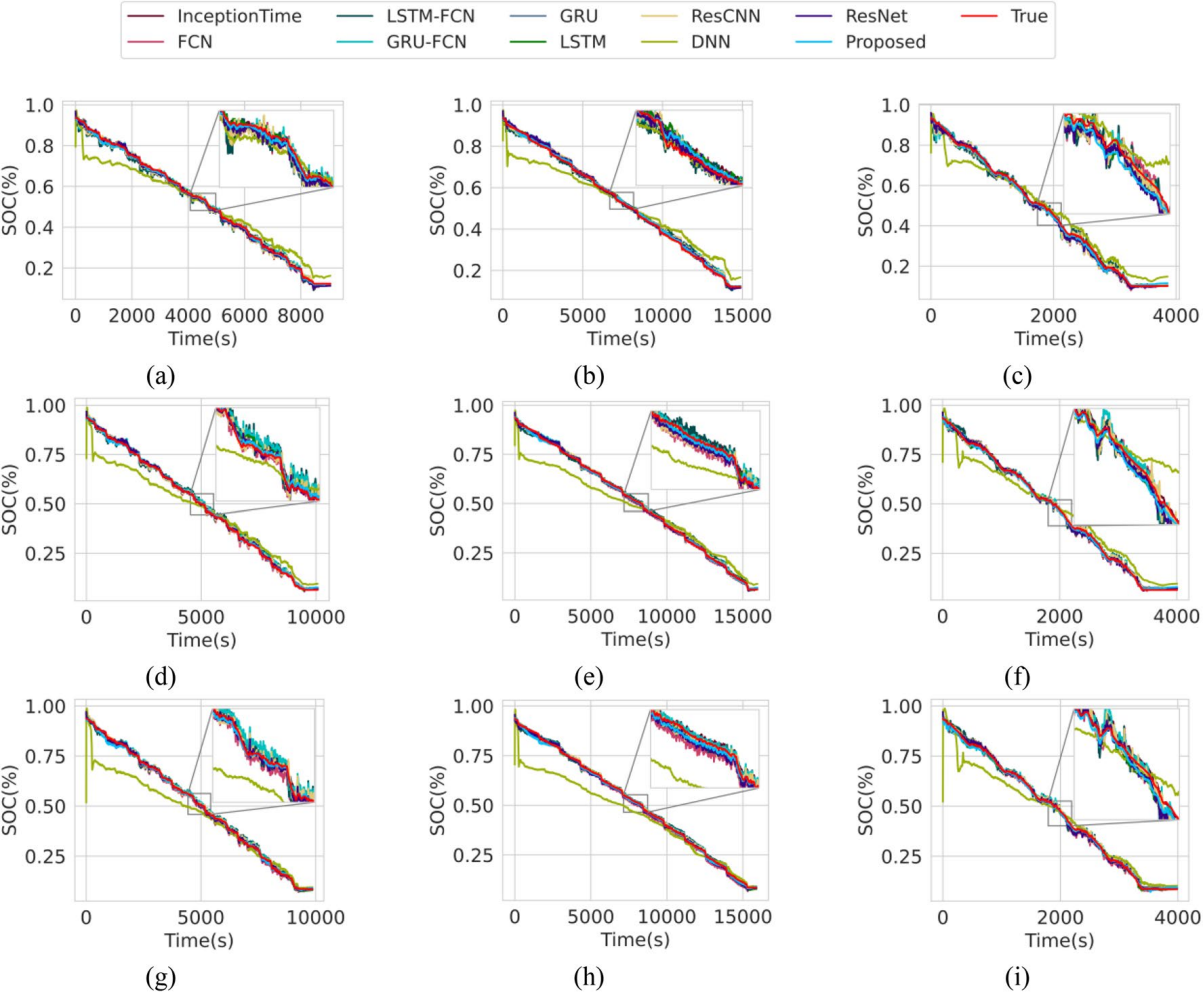
SOC estimation at above zero ambient temperatures. (**a**) LA92 drive cycle at 10 °C. (**b**) UDDS drive cycle at 10 °C. (**c**) US06 drive cycle at 10 °C. (**d**) LA92 drive cycle at 25 °C. (**e**) UDDS drive cycle at 25 °C. (**f**) US06 drive cycle at 25 °C. (**g**) LA92 drive cycle at 40 °C. (**h**) UDDS drive cycle at 40 °C. (**i**) US06 drive cycle at 40 °C.

**Figure 3:**
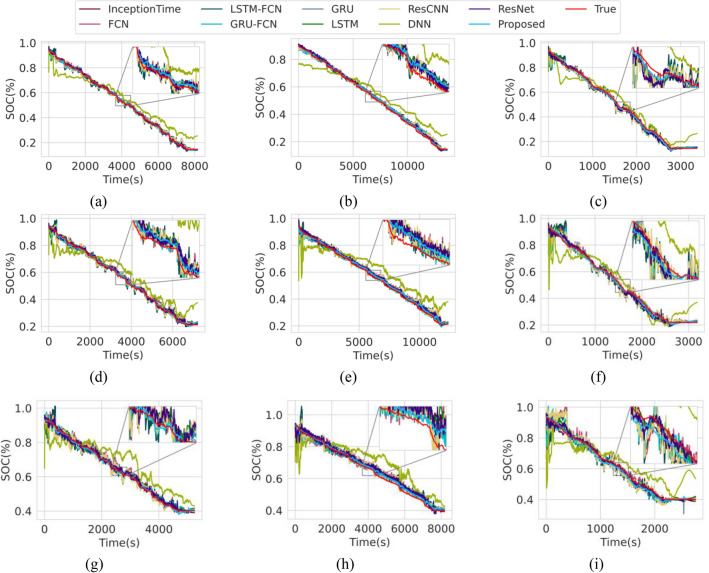
SOC estimation at below zero ambient temperatures. (**a**) LA92 drive cycle at 0 °C. (**b**) UDDS drive cycle at 0 °C. (**c**) US06 drive cycle at 0 °C. (**d**) LA92 drive cycle at − 10 °C. (**e**) UDDS drive cycle at − 10 °C. (**f**) US06 drive cycle at − 10 °C. (**g**) LA92 drive cycle at − 20 °C. (**h**) UDDS drive cycle at − 20 °C. (**i**) US06 drive cycle at − 20 °C.

In this subsection, we compare the estimation accuracy of the proposed model to other widely used DL models of various architectures. Among all DL architectures compared in the study, the proposed transformer model achieved the lowest RMSE of 1.1075%, 1.3139% and 1.1914% and MAE of 0.4441%, 0.5680% and 0.6502% on the test drive cycles outperforming even the recurrent models which has been widely used for SOC estimation as shown in Table 2. We also note that the convolutional models such as the Resnet^40^ and the Inception Time^51^ also outperformed the conventional GRU^41^ and LSTM^52^ model. The baseline Transformer model that is not trained with the proposed training framework scores poorly along with the feedforward DNN. Figure 2 and Fig. 3 illustrate the SOC estimation plots of the proposed model across all test drive cycles at above and below zero ^*◦*^C ambient temperature, respectively. Traditionally, SOC estimation under low ambient temperature settings are extremely challenging due to the difference in the dynamics of chemical reactions in the cell^53^. However, observation in the estimated SOC by the proposed Transformer model shows promising results indicating its robustness in estimating SOC at extreme cold temperatures up to − 20 °C.

**Table 2:** Cross-comparison of the SOC estimation accuracy on the train, validation, and test dataset at varying ambient temperatures.

Name	Arch. type	Train error (%)		Valid. error (%)		Test error (%)	
		RMSE	MAE	RMSE	MAE	RMSE	MAE
**Proposed**	**Transformer**	1.1075	0.4441	1.3139	0.5680	1.1914	0.6502
Resnet	Convolutional	1.2510	0.5077	1.5736	0.8058	1.3636	0.7771
InceptionTime	Convolutional	1.2112	0.4967	1.2573	0.5985	1.3792	0.8152
GRU	Recurrent	1.4183	0.3505	1.5989	0.4901	1.3856	0.5847
LSTM	Recurrent	1.5549	0.5681	1.5121	0.5961	1.4498	0.7300
ResCNN	Convolutional	1.5803	0.7712	1.8668	0.9860	1.5860	0.9048
GRU-FCN	Hybrid	1.5903	0.7695	2.0070	0.9893	1.6215	0.9269
FCN	Convolutional	1.7358	0.8879	2.1282	1.1033	1.7808	1.0810
LSTM-FCN	Hybrid	1.9962	1.0561	2.2292	1.1804	1.9248	1.1552
Baseline	Transformer	3.7466	2.4466	4.4805	3.4827	3.6958	2.8129
DNN	Feedforward	8.9848	6.8349	8.1916	6.1570	7.7789	6.1567

### Influence of pre-training

#### Size of training data

In this section, we investigate the influence of unsupervised pre-training on the amount of required data to train the proposed model with a low error rate. We divided the experiment into three scenarios. In the first scenario, we pre-trained and re-trained/fine-tuned the model with all (100%) available training data. The second and third scenarios were with 50% and 20% of the training data, respectively. In all scenarios, we noted the training time and error metrics. All training in this section was only performed for only 5 epochs to illustrate the short amount of training time required to achieve low error rates. There were three modes of training used in this setup namely the pre-training (PT), re-training (RT), fine-tuning (FT), and full training (T). In PT, the model was trained on unlabeled dataset with unsupervised learning. In RT, the mode was trained on a labeled dataset with supervised learning. In FT, the all the weights of the model were frozen except for the last layer and trained with supervised learning. In T, the model was trained from scratch with supervised learning.

Table 3 shows the results obtained. We observe that when we pre-trained and re-trained the model on all available data (row 1), the error metric is lower compared to the model that was not pre-trained (row 3). Both took approximately the same duration of training time. We observe that in the event where we pre-trained and fine-tuned the model (row 2), even though the model only updates the weights of the final layer, it still scores a respectable 2.10% test RMSE with a reduction of about 10 min training time compared to the previous two modes. The effect of pre-training is even more pronounced in the second scenario where we only re-trained and fine-tuned the models on 20% of train data. At approximately the same amount of training time, the pre-trained model (row 7) scores lower on the non-pre-trained model (row 9). In this section we show that pre-training helps in reducing the test error with approximately same amount of training time especially when there is very little training data.

**Table 3:** The influence of pre-training on the training time, training data amount, and cross-dataset generalization.

Mode*	Train time (mins)	Train data (%)	Train error (%)		Valid. error (%)		Test error (%)	
			RMSE	MAE	RMSE	MAE	RMSE	MAE
PT + RT	33.1	100	1.4378	0.7229	1.6207	0.8074	1.4257	0.8384
PT + FT	20.8	100	2.2281	1.4314	2.5211	1.4666	2.1042	1.4907
T	32.0	100	1.6981	0.9980	1.8448	1.1185	1.6367	1.0675
PT + RT	15.3	50	1.4318	0.6980	1.6749	0.9305	1.6578	1.0609
PT + FT	10.0	50	2.4540	1.6110	2.8765	1.7509	2.3893	1.7097
T	15.3	50	2.6117	1.7230	2.5167	1.5975	2.3953	1.7047
PT + RT	6.7	20	1.3072	0.6778	2.7138	1.3145	1.8993	1.1944
PT + FT	5.3	20	2.1391	1.4582	3.0693	1.8374	2.7173	1.9036
T	6.8	20	2.4656	1.5721	2.8898	1.7170	2.5900	1.7833

*PT Pre-training, *RT* Re-training, *FT* Fine-tuning, *T* Training.

#### Transfer learning

In this section we investigate the role of unsupervised pre-training in helping the model to generalize its estimation capacity across different cell chemistry. We pre-trained the model on the LG 18650 LiNiMnCoO_2_ cell and tested the model’s estimation capacity on a Panasonic 18650 LiNiCoAlO_2_ cell, similar to the cells used in some Tesla vehicles. Table 4 shows the performance of the proposed model with no changes in the model architecture.

**Table 4:** Cross dataset performance.

Mode*	PT dataset	RT/FT dataset	Train data (%)	Train error (%)		Valid. error (%)		Test error (%)	
				RMSE	MAE	RMSE	MAE	RMSE	MAE
PT + RT	LG	Panasonic	100	1.3128	0.7948	1.4245	0.8266	2.3558	1.5477
PT + FT	LG	Panasonic	100	1.9400	1.2514	2.4220	1.4254	3.3119	2.3490
T	–	Panasonic	100	1.2744	0.8086	1.4597	0.9165	2.7054	2.0383
PT + RT	LG	Panasonic	50	1.1674	0.6602	1.5817	0.9911	3.0465	2.1906
PT + FT	LG	Panasonic	50	2.0930	1.3643	2.9021	1.7583	3.8216	2.6578
T	–	Panasonic	50	1.4121	0.9128	1.8295	1.1583	3.1793	2.3900
PT + RT	LG	Panasonic	20	1.2377	0.7689	2.6921	2.0752	3.7735	2.8997
PT + FT	LG	Panasonic	20	1.9597	1.2957	3.4970	2.4140	3.8079	2.6289
T	–	Panasonic	20	1.5928	1.0598	3.1262	2.0098	4.3440	2.6932
PT + RT	LG	LG	100	9.4881	8.6845	9.7792	8.9473	11.3084	10.4053
PT + RT	Panasonic	Panasonic	100	1.0752	0.5757	1.3006	0.7778	2.3462	1.6941

*PT Pre-training, *RT* Re-training, *FT* Fine-tuning, *T* Training.

**Figure 4:**
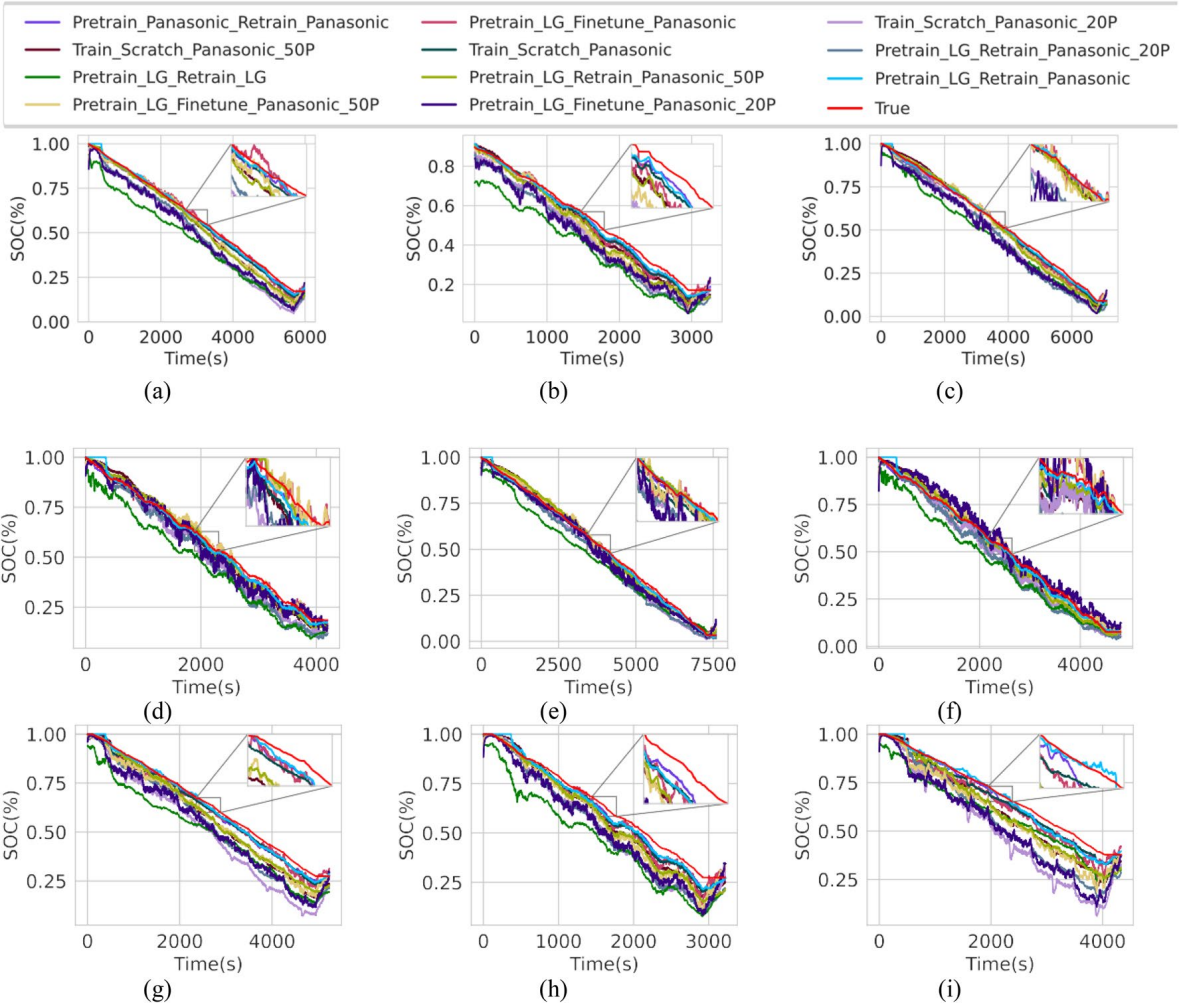
Estimation plot on the test drive cycles of the Panasonic cell with various training combinations. (**a**) HWFET cycle at 0 °C. (**b**) US06 cycle at 0 °C. (**c**) HWFET cycle at 10 °C. (**d**) US06 cycle at 10 °C. (**e**) HWFET cycle at 25 °C. (**f**) US06 cycle at 25 °C.

We divided the experiment into four scenarios. In the first scenario, we pre-trained and re-trained/fine-tuned the model with all (100%) available training data. The second and third scenarios were with 50% and 20% of the training data, respectively. In the fourth scenario we pre-trained and re-trained the model on the same cell type with all available data shown in the last two rows of Table 4. Unsurprisingly, the best performing mode is when the model pre-trained and re-trained on the Panasonic cell and the worst performing model is when the model was pre-trained and re-trained on the LG cell. However, when the model was pre-trained on the LG cell and re-trained on the Panasonic cell, the test error rate is almost on par with the best performing model. This suggests that the pre-training helps in downstream re-training despite the difference in cell type. In the scenario when the model was trained on less data (20% of the training data), we observe that without pre-training (row 9) the model yields high test errors. In rows 7 and 8, we observe that the error rate is reduced by pre-training the model, even on a different cell. This once again is evidence that pre-training contributes to minimizing the test set error regardless of the cell type. Supplementary Fig. 2 illustrates the estimation of the worst performing mode. Despite being trained on a different cell type, the model still can capture the trend of the ground truth SOC value. With pre-training on the LG cell and re-training on the Panasonic cell, model can estimate the SOC more accurately as shown in Fig. 4.

In this section, we showed that the weights of the model that is learned during the unsupervised pre-training phase can be reused in re-training or fine-tuning across different cell types. This opens the possibilities of transfer learning which is extremely helpful especially when data and computational resource is scarce. On a side note, all re-training and fine-tuning in this and the previous section was only performed for 5 epochs to showcase the learning capability of the model despite a small training epoch. Re-training and fine-tuning the model for more epochs will likely yield better performance.

## Discussion

In this work, a Transformer-based SOC estimation model in combination with the SSL framework was developed to address the challenges on Li-ion cell data availability, transfer learning, training speed and model accuracy. We show that the proposed model can achieve the lowest RMSE and MAE on the test set at various ambient temperature settings. The proposed technique also enables the Transformer model to be trained in a relatively short amount of time. The first contribution of this work is the introduction of a novel Transformer DL architecture that is capable of accurately estimating the SOC of a Li-ion cell under constant and varying ambient temperatures. Based on the provided dataset, the model can accurately estimate the SOC up to RMSE ≤ 1.19% and MAE ≤ 0.65% (at varying ambient temperatures) and RMSE ≤ 0.9% and MAE ≤ 0.44% (at constant ambient temperature) with no feature engineering or any type of filtering. This also shows that the transformer can self-learn the model parameters and map the voltage, current and temperature input data directly to SOC.

The second contribution is the self-supervised learning (SSL) training scheme to effectively train the proposed model. Even though the conventional supervised learning (SL) scheme can train the proposed model up to a reasonably low error (RMSE ≤ 1.63% at varying ambient temperatures), this work highlights that the SSL training framework is advantageous in further reduction of error rate (RMSE ≤ 1.42% at varying ambient temperatures) at approximately the same amount of training time. This suggests that the SSL framework proposed to train the Transformer contributes to lowering the RMSE.

The third contribution of this work is to demonstrate that the weights from the encoder layers of the Transformer learned using the unsupervised pre-training phase can be readily transferred to another cell type of a different chemistry. Additionally, with only five epochs of re-training, the model can achieve RMSE ≤ 2.5% on the test set. Extending the training time further likely leads to further reduction in RMSE. However, this work shows that even with lightweight re-training, the weights transferred from pre-training significantly contribute to the short training time with significantly less data. This opens the possibility of adapting the Transformer model to other types of Li-ion cell with only a fraction of the training data.

The fourth contribution highlights the SSL training framework that enables the proposed model to be re-trained with as few as 20% of the total training data and still achieve lower error compared to the models that do not use the SSL framework. Despite the reduced amount of training data, the model still generalizes well across different cell chemistry. This further accentuates the important role of unsupervised pretraining in allowing the model to be more data efficient.

The fifth contribution compares and validates the performance of the model against recent state-of-the-art DL models on SOC estimation. It is shown that the model clearly outperforms all other models in the RMSE and MAE metric on the test dataset. Given the efficacy of the model in SOC estimation accuracy, transfer learning capability, data-efficiency and training speed, the proposed Transformer model and framework is evidently advantageous over other DL models.

## Methods

### Dataset

**Figure 5:**
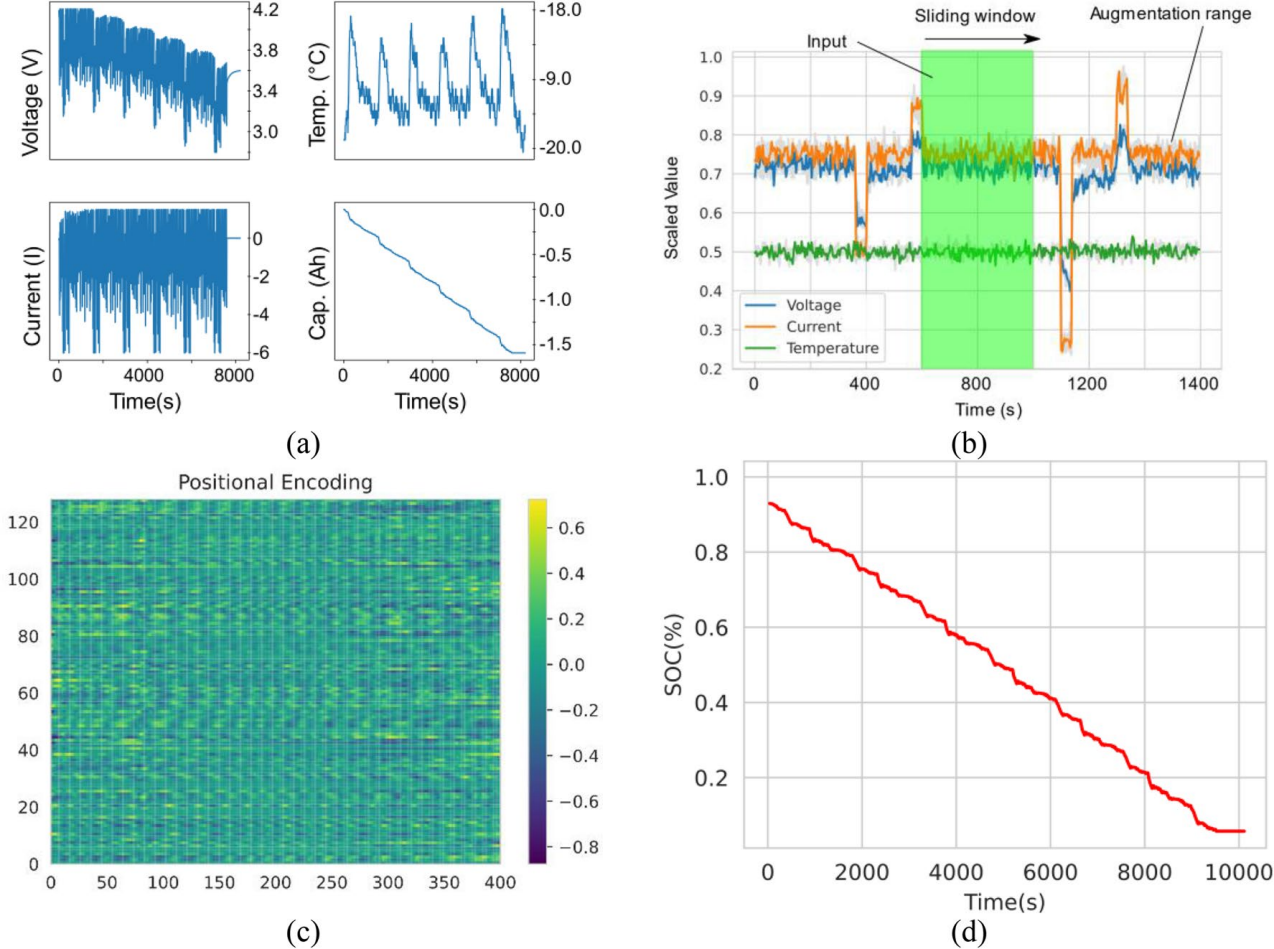
Data preprocessing pipeline from raw values to positional encoding. (**a**) Raw data sample plot from the UDDS drive cycle at − 20 °C ambient temperature. (**b**) Input-label pairs constructed by sliding a fix width window across the train, validation, and test dataset. (**c**) Dataset converted into positional encoding to be ingested into Transformer.

In this study, we utilized raw data sampled from a brand-new cylindrical 18,650 LiNiMnCoO2 cell by LG which was made available by the McMaster University in Hamilton, Ontario, Canada^54^. The specification of the cell is given in Supplementary Table 1. The data was collected by subjecting the battery cell to various EV standard drive cycles such as UDDS, HWFET, LA92, US06 at varying ambient temperatures ranging from − 20 to 40 °C. In addition, to simulate the dynamics of driving conditions, the cell was also subjected to a random mix of the standard drive cycles. The division of train, validation and test dataset used in this study is specified in Supplementary Table 3. Figure 5a illustrates a sample plot of the UDDS drive cycle from the test dataset at − 20 °C ambient temperature. For DL models to work well, careful consideration is put in preprocessing the raw data samples. Firstly, the raw data is normalized into a range of 0 to 1 using Eq. (1).

1 $$\tilde{x }=\frac{x-min\left(x\right)}{max\left(x\right)-min\left(x\right)}$$

Next, the raw data was divided into three separate sets namely train, validation, and test set. The train set was used to train the model, validation set to check the generalization of the model during training and the test set was only used to evaluate the model at the end of training. The division of the train, valid and test set is tabulated in Supplementary Table 3.

Using supervised learning requires the dataset to be formatted into input-label pairs. In this study the input was the normalized values of voltage, current, and temperature while the label corresponds to the SOC value. The input-label pairs were constructed by running a sliding window across the time axis over the train, validation, and test set as illustrated Fig. 5. Voltage, current and temperature values that resides in the green window corresponds to the input and SOC value that resides in the purple window corresponds to the label. Note that the width of the window, *k* was kept at *k* = 400 timesteps.

Having massive amounts of data is a crucial component in training the proposed model without which the model will fail to generalize well^55^. Although the raw data already consists of hundreds of thousands of timesteps, it is still insufficient for the proposed model to work. Hence, the original dataset was augmented in various way by injecting random noise (*µ* = 0, *σ* = 0*.*33) onto the training and validation dataset. The types of noise injected includes additive and multiplicative Gaussian noise on the magnitude and random frequency noise generated with the wavelet decomposition method. Figure 5b shows a sample of the original and the augmented version of the plot.

### Transformer model architecture

**Figure 6:**
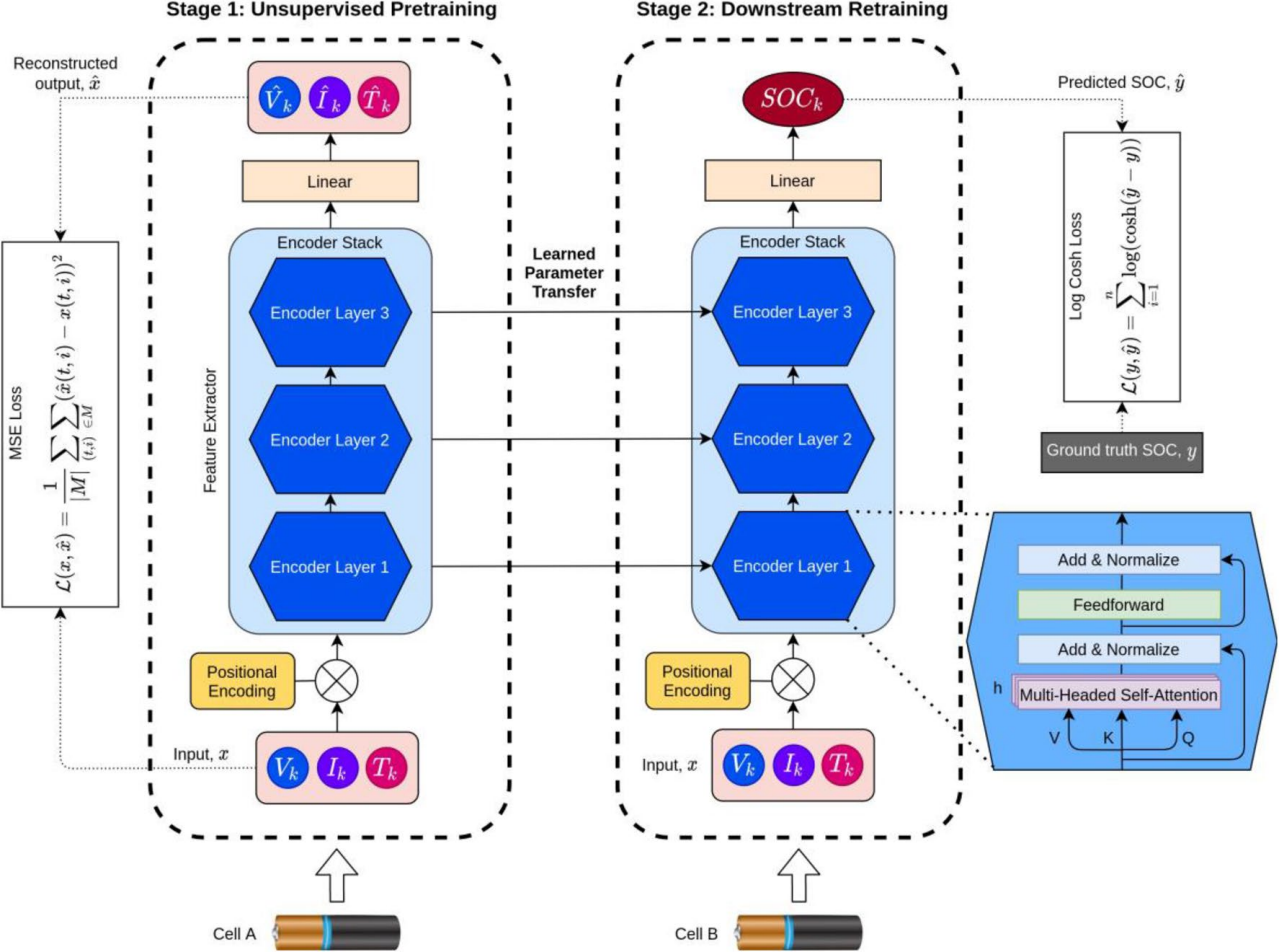
Framework and architecture of the proposed transformer model.

The original Transformer proposed in^56^ uses the encoder-decoder arrangement in the architecture. The model proposed in this work only adopts the encoder portion and not the decoder as detailed in^57^ to work better with multivariate time-series data. Figure 6 illustrates the block diagrams of the proposed model depending on the training stage which will be detailed more in the next section. Observe that in Stage 1 and Stage 2 there are several common blocks namely, input, x positional encoding, encoder stack, and linear layer. The input data to the model consists of the input vector of *X* = [*V*_*k*_*, I*_*k*_*, T*_*k*_] representing the cell voltage, current and temperature at timestep, *k*. To give the input data contextual information, the input vector is then added to the positional encoding. There are various choices of positional encodings according to^58^. However, in this work we use the learned positional encoding with the sine and cosine functions as shown in Eqs. (2) and (3).

2 $$P{E}_{\left(pos,2i\right)}=\mathrm{sin}\left(\frac{pos}{{10000}^{2i/{d}_{model}}}\right)$$

3 $$P{E}_{\left(pos,2i+1\right)}=\mathrm{cos}\left(\frac{pos}{{10000}^{2i/{d}_{model}}}\right)$$

where *pos* and *i* correspond to the position and dimension, respectively. The reasoning behind using both functions has been previously detailed in^56^. Once tagged with the positional encodings, the input vector is passed through a series of encoder blocks. Core to the Transformer architecture is the multi-headed self-attention (MHSA) module inside the encoder block. The MHSA module applies self-attention to the input sequence with respect to the output sequence. As shown in the figure, the input to the multi-headed self- attention module are the key, *K*, value, *V* and query, *Q*. In the MHSA block, it attempts to map the query to a set key-value pairs with respect to an output to produce the attention matrix. The operation consists of a dot product of the query, Q with all keys and a division by *d*_*k*_ and applying a softmax function over the result as given in Eq. (4) where dk is the dimension of the keys.

4 $${\text{Attention}}\left(Q,K,V\right)={\text{softmax}}\left(\frac{Q{K}^{T}}{\sqrt{{d}_{k}}}\right)V$$

Instead of performing the operation in Eq. (4) once to produce a single matrix, the operation can be repeated multiple times in parallel and the resulting matrices can be concatenated into a larger matrix as show in Eq. (5).

5 $${\text{MultiHead}}\left(Q,K,V\right)={\text{Concat}}\left(Attentio{n}_{1},Attentio{n}_{2}\dots Attentio{n}_{n}\right){W}^{O}$$

In this work we use the number of parallel attention heads, *h* = 16. Each head uses *d*_*model*_ = 128. Also used in the encoder module is the residual connection and residual dropout, which was set at dropout probability, *p* = 0*.*1.

The remaining model hyperparameter values are in Supplementary Table 4. Since Transformer models processes the entire sequence and does not account for the order of the input, a positional encoder is required to add contextual information. The positional encoder generates a unique encoding for each data point in the input vector and can generalize to longer sequences. Shown in Fig. 5c is the learned positional encoding generated for the input dataset in this study which consists of voltage, current and temperature of the Li-ion cell. The x-axis corresponds to the lag window in the input dataset which is used generate the dataset.

### Training framework

Instantiating a DL model involves various stochastic processes. To ensure the reproducibility and consistency of the results obtained, all experiments were conducted using a preset seed value. Referring to Fig. 6, model training was divided into two distinct phases, namely the unsupervised pre-training and downstream fine-tuning. In the unsupervised pre-training stage^59^, unlabeled vectors of input sequence, X was used to train the model. Part of each input sequence values were randomly set to 0 by performing element-wise multiplication with a binary mask, M. The corrupted input, X˜ was generated with the X˜ = M 0 X. The model was then required to reconstruct the masked input with a modified MSE loss function, as given in Eq. (6).

6 $$\mathcal{L}\left(x,\widehat{x}\right)=\frac{1}{\left|M\right|}{\sum }_{\left(t,i\right)}{\sum }_{\in M}{\left(\widehat{x}\left(t,i\right)-x\left(t,i\right)\right)}^{2}$$

where $\widehat{x}$ is the predicted input vector values and *x* is the un-corrupted input vector values. Note that the loss does not require the model to reconstruct the entire input sequence but only elements in the mask, *M*. Upon completion of the unsupervised pre-training phase, the weights of the model save were transferred for the downstream fine-tuning phase. In this phase, the model was re-trained on a labeled dataset with supervised learning. The loss function, used in this phase is the hyperbolic cosine (Log-cosh) loss function as given in Eq. (7).

7 $$\mathcal{L}\left(y,\widehat{y}\right)={\sum }_{i=1}^{n}\mathrm{log}\left(\mathrm{cosh}\left(\widehat{y}-y\right)\right)$$

where *y is* the ground truth and *y*ˆ is the predicted value by the model. The RMSE [Eq. (8)] and MAE [Eq. (9)] error metric was used to evaluate all models.

8 $$\mathrm{RMSE}=\sqrt{\frac{1}{\mathrm{N}}{\sum }_{\mathrm{k}=1}^{\mathrm{N}}{\left({\mathrm{SOC}}_{\mathrm{k}}-{\mathrm{SOC}}_{\mathrm{k}}^{*}\right)}^{2}}$$

9 $$MAE=\frac{1}{N}{\sum }_{k=1}^{N}\left(\left|SO{C}_{k}-SO{C}_{k}^{*}\right|\right)$$

One of the most important hyperparameter used in training DL models is the learning rate, *α* (LR)^60^. To search for the optimal LR range of values, we employ the use of LR finder introduced in^61^. The optimal LR found with the LR finder is *α* = 1*e*3 as depicted in Supplementary Fig. 2. The LR value was used in conjunction with the Ranger optimizer which is a synergistic combination of Rectified Adam (RAdam)^62^ and Lookahead optimizer^63^. RAdam has been shown to stabilize the training at the start and Ranger stabilizes the convergence in the remaining steps^64^. The Ranger optimizer is configured with momentum = 0.95, weight decay = 0.01 and epsilon of 1e^−6^. This combination has been shown to achieve state-of-the-art results on many datasets^65,66^. As the training approaches the end, the LR is decayed to a lower value to further facilitate convergence to a global minimum^67^. The LR is decayed for each batch as follows,

10 $${\eta }_{t}={\eta }_{min}^{i}+\frac{1}{2}\left({\eta }_{max}^{i}-{\eta }_{min}^{i}\right)\left(1+\mathit{cos}\left(\frac{{T}_{current}}{{T}_{i}}\pi \right)\right)$$

*η* is the maximum and minimum LR values, and *Tcurrent* is the number of epochs since the last restart. Figure shows the LR values throughout the training. The training hyperparameter values of the proposed Transformer model is concisely summarized in Supplementary Table 5.

### Implementation

All models studied were trained on an Ubuntu 20.04.02 LTS Linux operating system with Intel Core i7-4790 K CPU at 4.00 GHz clock frequency, 32 GB of RAM and a Nvidia GeForce RTX3090 graphic processing unit. All DL models were built using the open source Pytorch 1.7.1^68^ framework in tandem with the TSAI library^69^. The implementation of the proposed Transformer model and SSL training framework was divided into several steps. In Step 1, two distinct datasets from the LG *LiNiMnCoO*2 cell (Supplementary Table 1) and Panasonic *LiNoCoAlO*2 cell (Supplementary Table 2) were downloaded. Both dataset consist of data sampled from respective cells over a diverse range of temperature and drive cycles to simulate dynamic operating conditions as elaborated in Sect. 3.1. The dataset was divided into train, validation and test sets as shown in Supplementary Table 3. Next the data was normalized into the appropriate range (0 to 1) and pre-processed with sliding window of lag, *k* = 400 timesteps (Fig. 5b). The sliding window lag value, *n* is arbitrarily selected to due to the limits in our computing resources. Given more computational resources, *k* can be made larger to allow the model to consider more contextual information from the past. At this point the dataset was also augmented by injecting additive and multiplicative Gaussian noise. Finally, the dataset is transformed into the positional encoding form shown in Fig. 5c. This is the format of the data that is expected by the transformer model.

In Step 2, the Transformer was configured using the appropriate model hyperparameters as detailed in Supplementary Table 4. Careful attention is placed on the dropout hyperparameter value as it largely influences the degree of overfitting on the dataset. We find that the settings of dropout in the feedforward layer to *p* = 0.2 and dropout in the residual layer to *p* = 0.1 work well in our experiments.

In Step 3, the model is now ready to be trained. As illustrated, the model was trained in two distinct stages in the order of unsupervised pretraining and then downstream retraining. In “Estimation accuracy under constant ambient temperature”, the model was trained on the LG dataset and was evaluated on its estimation accuracy at fixed and variable ambient temperature settings. In “Estimation accuracy under varying ambient temperatures.”, the model was trained on the LG dataset and tested on its estimation accuracy on the Panasonic dataset. In this step, the training hyperparameter was configured as detailed in Supplementary Table 5. Careful attention was placed on setting the LR in both training phase as it largely determines the performance and convergence to a global minimal. We rely extensively on the use of a LR finder algorithm which points us to setting the LR, *α* = 1 × 10^–3^ for pretraining and *α* = 2 × 10^–4^ for retraining.

In Step 4 the model was evaluated on the SOC estimation accuracy in “Estimation accuracy under constant ambient temperature” and the influence of pretraining and SSL on the performance of the proposed model in “Estimation accuracy under varying ambient temperatures.”. The performance of the model was quantified with the RMSE and MAE performance metrics. Finally, the performance of the model is compared to various widely used DL models on similar performance metrics.

## Supplementary Information

Supplementary Information.

## Data Availability

The data that support the findings of this study are available from the corresponding author upon reasonable request.

## Code availability

The software code and the examined cases that validated our method are available from the corresponding author upon reasonable request.
