# Supplementary material for: Deep learning approach towards accurate state of charge estimation for lithium-ion batteries using self-supervised transformer model
Source: Sci Rep. 2021 Oct 1;11:19541. doi: 10.1038/s41598-021-98915-8 (PMC8486825; doi:10.1038/s41598-021-98915-8)
Supplement: Supplementary file 1 — Supplementary Information. [file 41598_2021_98915_MOESM1_ESM.docx]

**Supplementary Information**

**Deep learning approach towards accurate state of charge estimation for lithium-ion batteries using self-supervised transformer model**

M A Hannan^1*^, Dickson N T How^1^, M S Hossain Lipu^2^, M. Mansor^1^, Pin Jern Ker^3^, Z Y Dong^4^, K S M Sahari^5^, S K Tiong^3^, K M Muttaqi^6^, T M Indra Mahlia^7^, F Blaabjerg^8^

^1^Department of Electrical Power Engineering, COE, Universiti Tenaga Nasional, Kajang 43000, Malaysia

^2^Dept of Electrical, Electronic and Systems Engineering, Universiti Kebangsaan Malaysia, Bangi 43600, Malaysia

^3^Institute of Sustainable Energy, Universiti Tenaga Nasional, Kajang 43000, Malaysia

^4^School of Electrical Engineering and Telecommunications, UNSW, Kensington NSW 2033, Australia

^5^Department of Mechanical Engineering, COE, Universiti Tenaga Nasional, Kajang 43000, Malaysia

^6^School of Electrical, Computer and Telecommunications Engineering, University of Wollongong, NSW 2522, Australia

^7^School of Information, Systems and Modelling, University of Technology Sydney, Ultimo NSW 2007, Australia

^8^Department of Energy Technology, Aalborg University, 9220 Aalborg, Denmark

*Corresponding author’s email: [hannan@uniten.edu.my](mailto:hannan@uniten.edu.my); [dickson@uniten.edu.my](mailto:dickson@uniten.edu.my)

**Supplementary Table 1:** Specifications of the cylindrical LG18650 *LiNiMnCoO*_2_ cell.

**Supplementary Table 2:** Specifications of the cylindrical Panasonic *LiNiMnCoO*_2_ cell.

**Supplementary Table 3:** Train, validation, and test set drive cycles.

**Supplementary Table 4:** Architecture hyperparameter values for model.

**Supplementary Table 5:** Training hyperparameter of the proposed Transformer model.

**Supplementary Fig. 1:** (a) Optimal region (in green shade) of learning rate values that corresponds to the most rapid decline in the loss value and hence are better suited to be used in training. (b) Learning rate schedule during the training process. (c) Loss values on the training and validation set during training. (d) RMSE metric on the training and validation set during training.

**Supplementary Fig. 2: (**a) Relation between training modes and the RMSE metric. (b) Relation between training modes and training time.

**Supplementary Table 1.** Specifications of the cylindrical LG18650 *LiNiMnCoO*_2_ cell.

| Type | Nominal  Capacity (Ah) | Nominal  Voltage (V) | Cut-off  Voltage (V) | Maximum  Current (A) | Specific  Energy (Wh/kg) |
| --- | --- | --- | --- | --- | --- |
| 18650 NMC | 3.0 | 3.6 | 2.5/4.2 | 20 | 240 |

**Supplementary Table 2.** Specifications of the cylindrical Panasonic *LiNiMnCoO*_2_ cell.

| Type | Nominal  Capacity (Ah) | Nominal  Voltage (V) | Cut-off  Voltage (V) | Maximum  Current (A) | Specific  Energy (Wh/kg) |
| --- | --- | --- | --- | --- | --- |
| 18650 NMC | 2.9 | 3.6 | 2.5/4.2 | 10 | 206 |

**Supplementary Table 3.** Train, validation, and test set drive cycles.

| Dataset | Dataset Drive Cycles | Total timesteps |
| --- | --- | --- |
| Train | Mix1, Mix2, Mix3, Mix4, Mix5, Mix6, HPPC, HWFET | 318,072 |
| Validation | Mix7, Mix8 | 68,032 |
| Test | UDDS, LA92, US06 | 152,215 |

**Supplementary Table 4.** Architecture hyperparameter values for model.

| Hyperparameter | Value |
| --- | --- |
| Input dimension | 3 (voltage, current, temperature) |
| Number of layers in encoder | 3 |
| Sequence length | 400 |
| Number of features | 128 |
| Type of positional encoder | Zeros |
| Number multiheaded attention heads | 16 |
| Batch normalization | False |
| Dimension of feedforward layer | 256 |
| Activation function | Gaussian Error Linear Units (GELU) |
| Dropout in feedforward layer | 0.2 |
| Residual dropout in encoder | 0.1 |

**Supplementary Table 5.** Training hyperparameter of the proposed Transformer model.

| Hyperparameter | Value | |
| --- | --- | --- |
| Pretraining LR | 1e-3 |  |
| Retraining LR | 2e-4 |  |
| LR Schedule | Flat and cosine annealing |  |
| Optimizer | Ranger |  |
| Minibatch size | 128 |  |
| Training epochs | 25 |  |
| Loss function | Log-cosh |  |
| Error metric | RMSE & MAE |  |

**Supplementary Figures**

Supplementary Fig. 1 (a) shows the optimal region (in green shade) of learning rate values that corresponds to the most rapid decline in the loss value and hence are better suited to be used in training. Supplementary Fig. 1 (b) shows the learning rate schedule during the training process. In the beginning 75% of the training loop, the learning rate is held at a constant value and during the last 25% of the training, the learning rate value is decayed following the cosine function. Supplementary Fig. 1 (c) shows the loss values on the training and validation set during training. Supplementary Fig. 1 (d) shows the RMSE metric on the training and validation set during training.

| 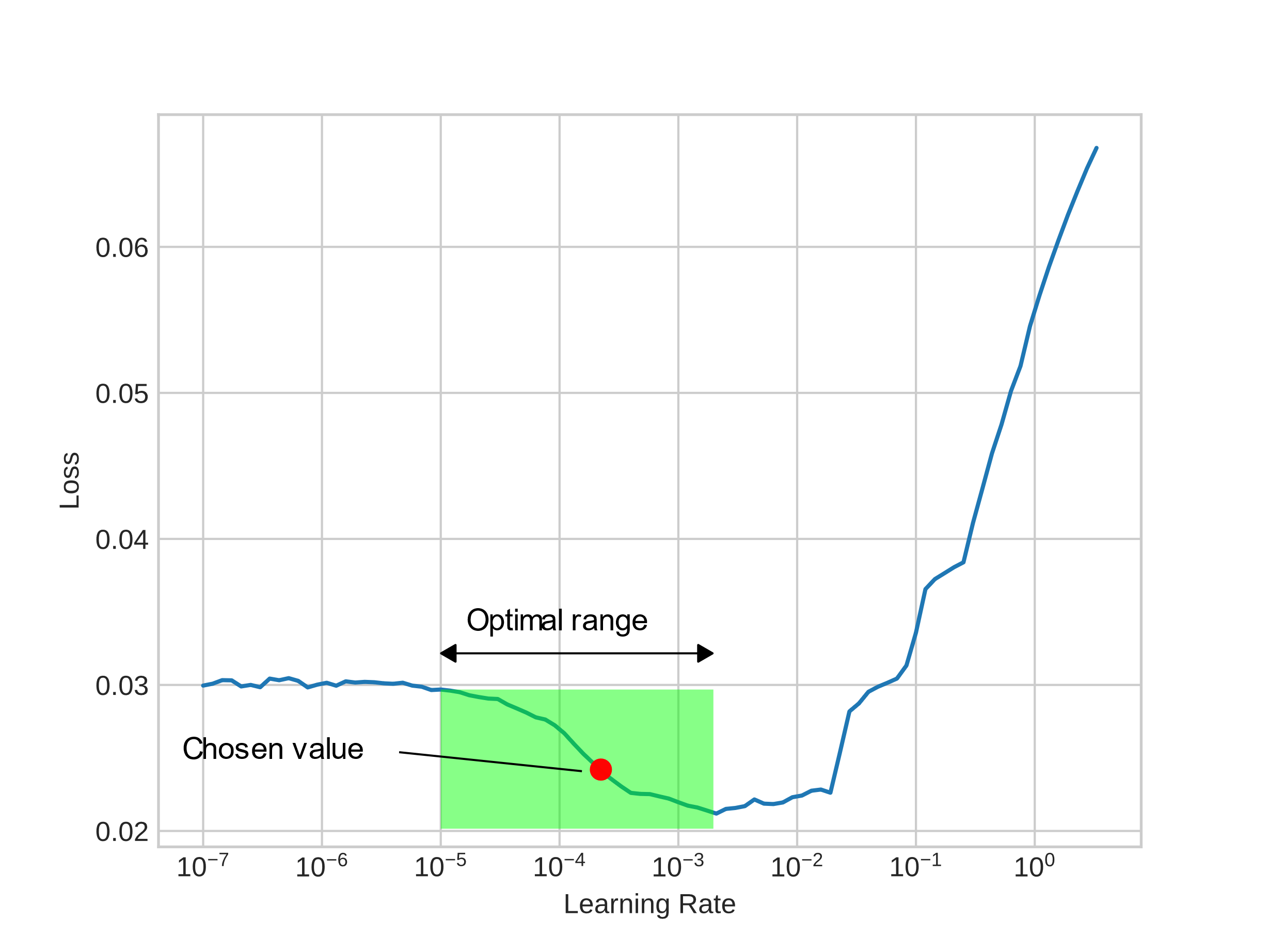 | 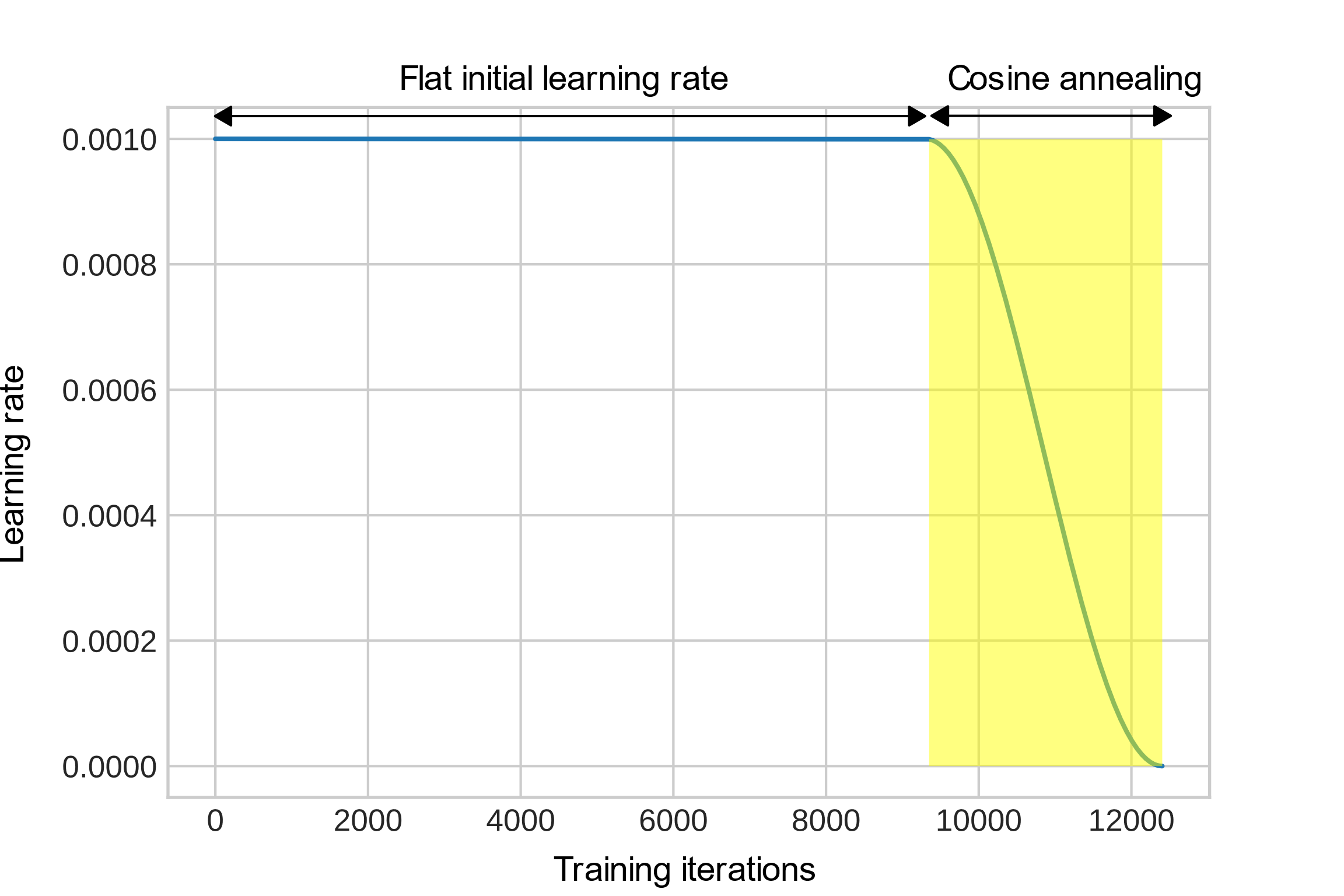 |
| --- | --- |
| (a) | (b) |
| 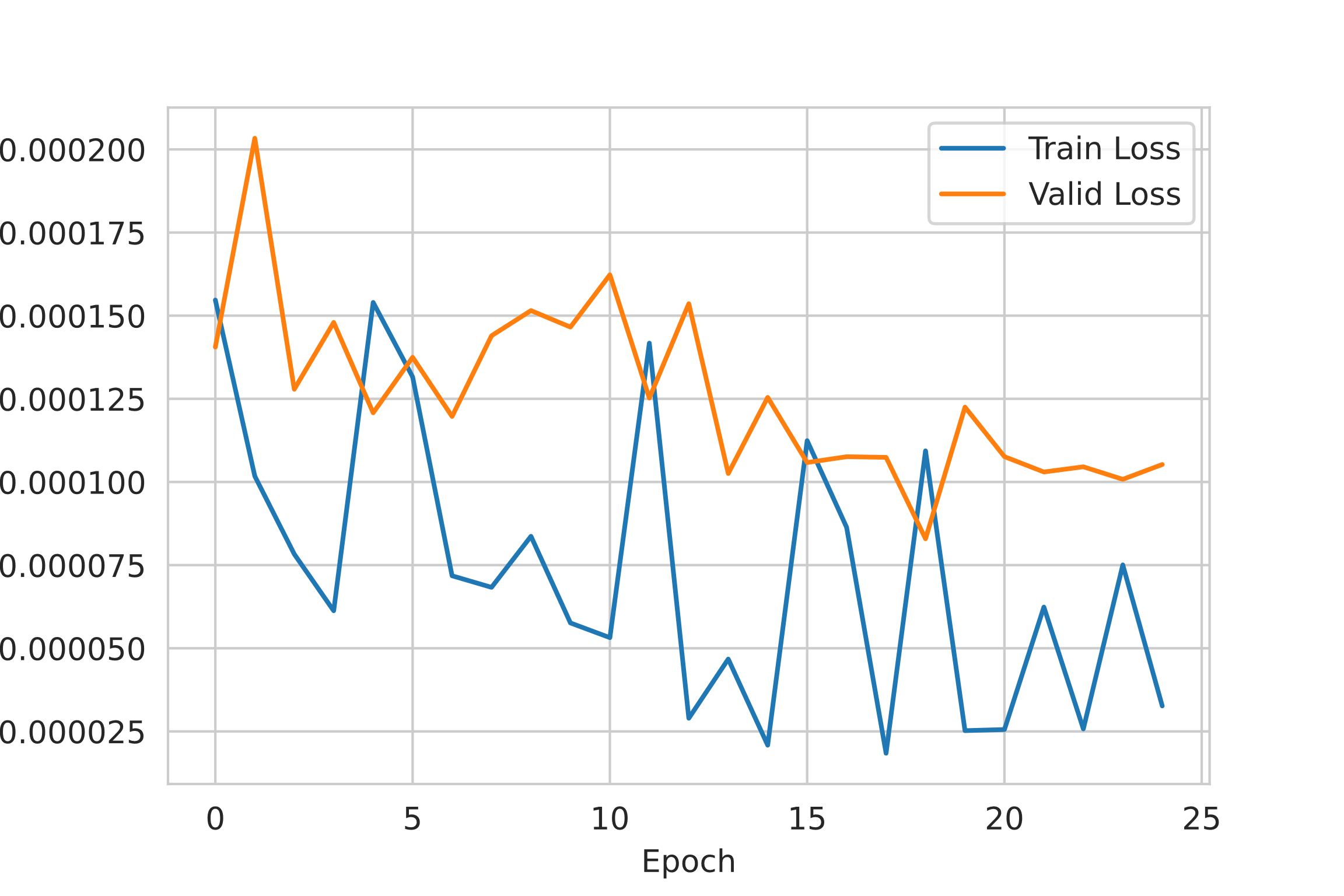 | 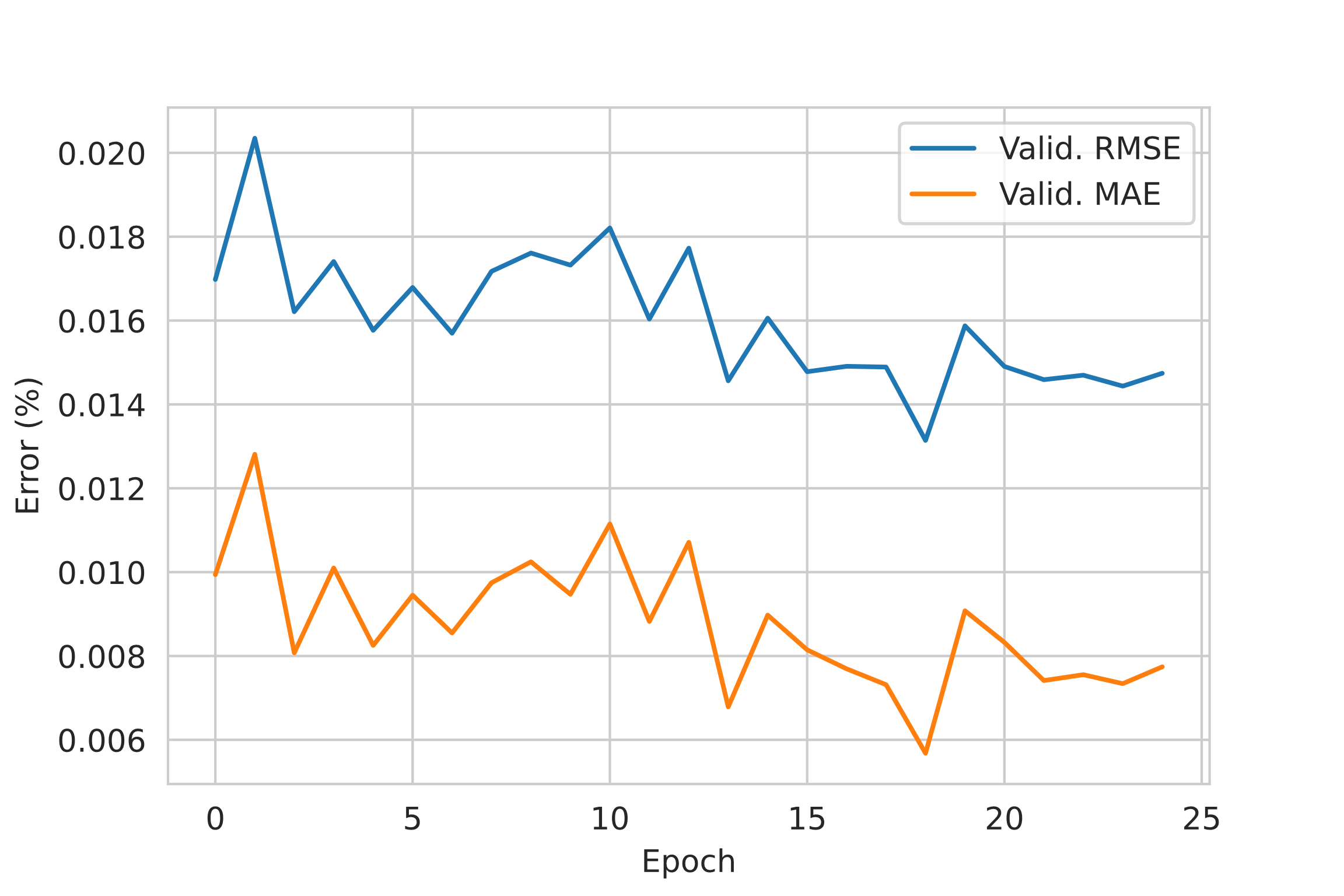 |
| (c) | (d) |

**Supplementary Fig. 1** (a) Optimal region (in green shade) of learning rate values that corresponds to the most rapid decline in the loss value and hence are better suited to be used in training. (b) Learning rate schedule during the training process. (c) Loss values on the training and validation set during training. (d) RMSE metric on the training and validation set during training.

Supplementary Fig. 2 (a) shows the relation between training modes and the RMSE metric and supplementary Fig. 2 (b) shows the relation between training modes and training time. Supplementary Fig. 2 (a) shows that PT+RT always results in lower RMSE compared to T and PT+FT. Supplementary Fig. 2 (b) shows that PT+RT takes almost the same amount of training time compared to training mode T. In both subfigures, it is evident that PT+RT mode contributes to the lowest RMSE and take approximately the same amount of time compared to models trained in training mode T.

| 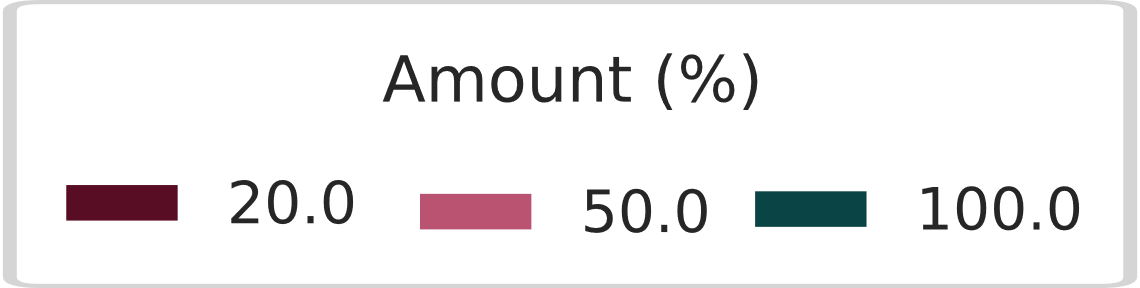 | |
| --- | --- |
| 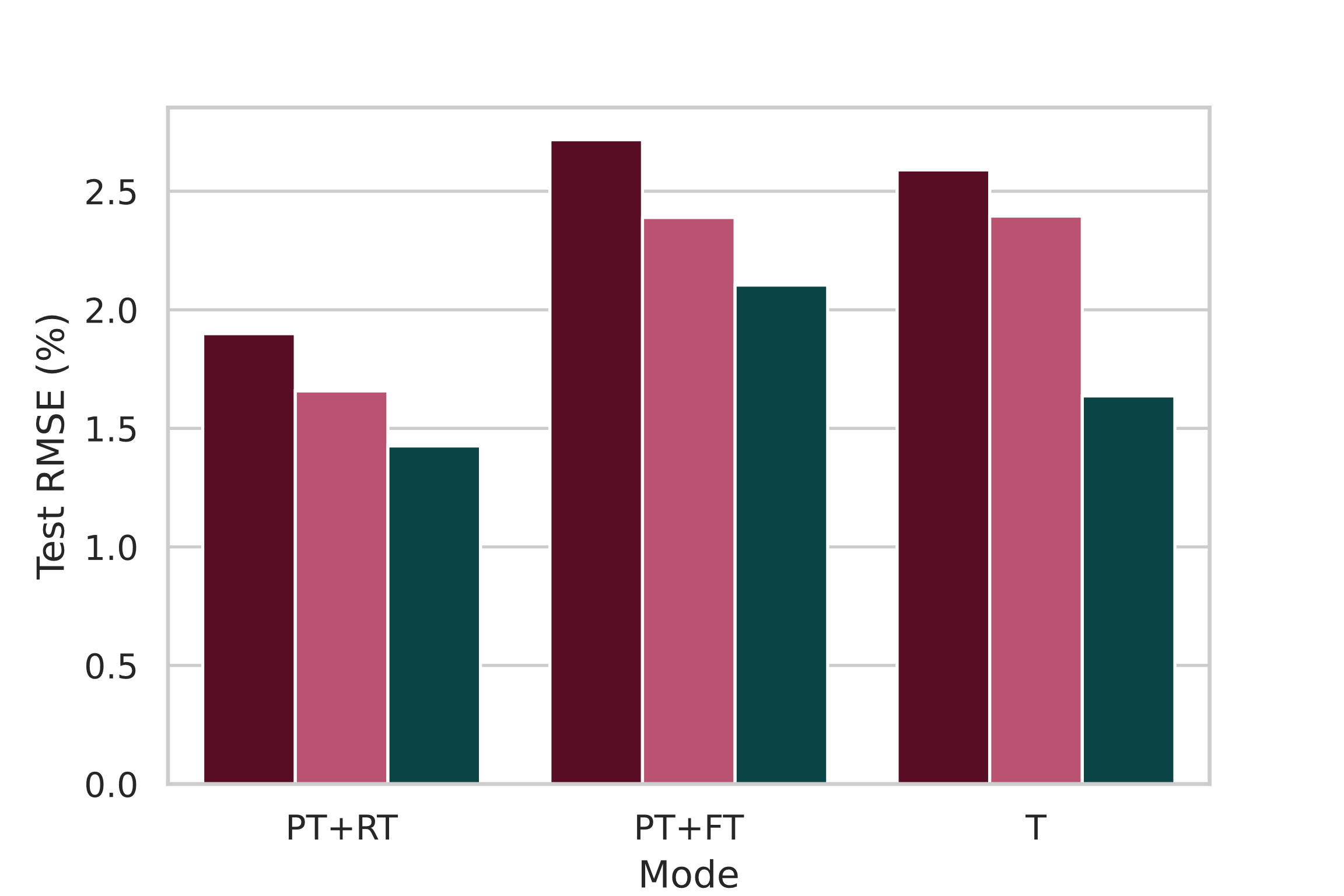 | 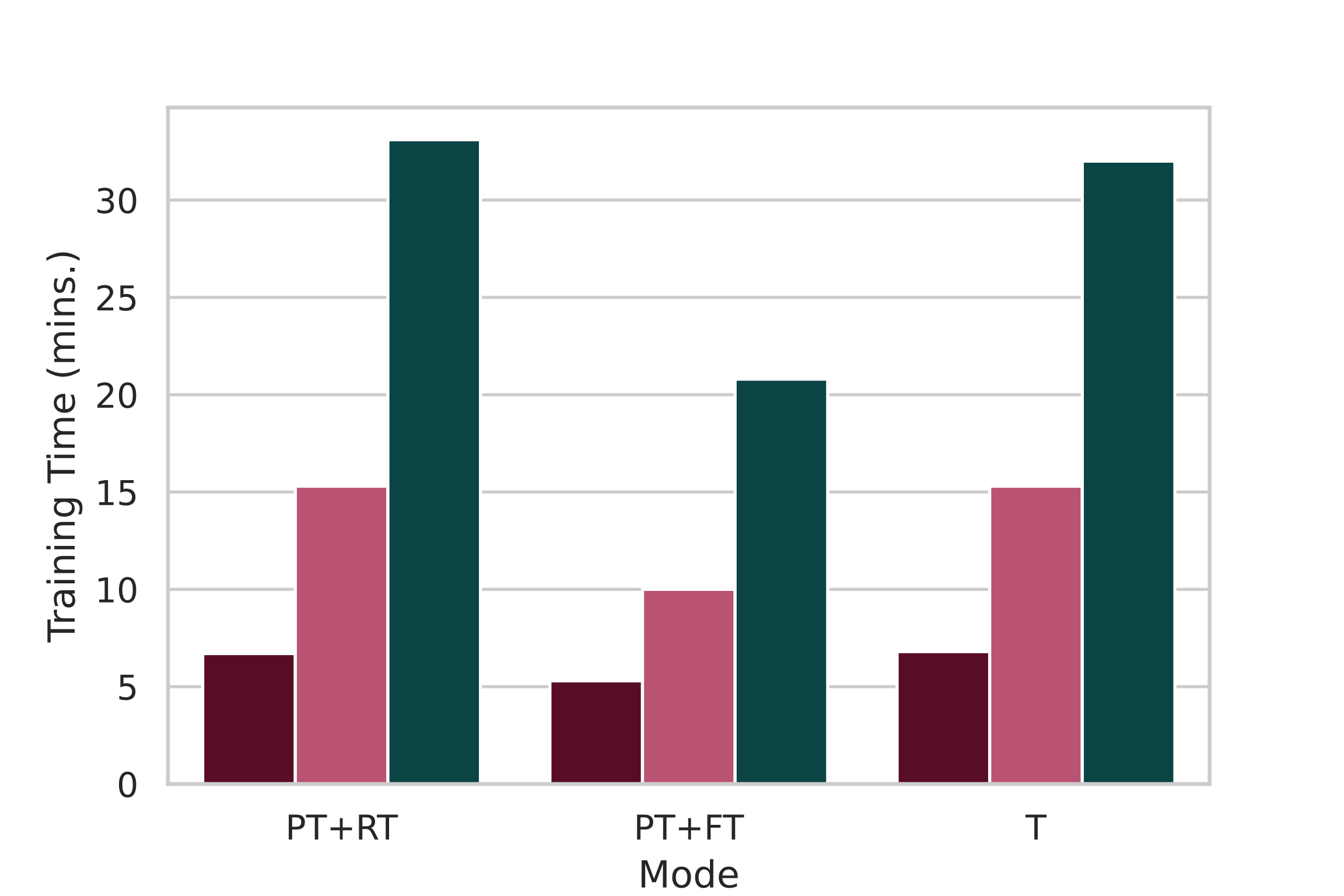 |
| (a) | (b) |

**Supplementary Fig. 2. (**a) Relation between training modes and the RMSE metric. (b) Relation between training modes and training time.
